# Supplementary material for: PyVaporation: A Python Package for Studying and Modelling Pervaporation Processes
Source: Membranes (Basel). 2022 Aug 15;12(8):784. doi: 10.3390/membranes12080784 (PMC9416308; doi:10.3390/membranes12080784)
Supplement: Supplementary file 1 [file membranes-12-00784-s001.zip › Experimental setup description.pdf]

Description of the pervaporation experimental setup

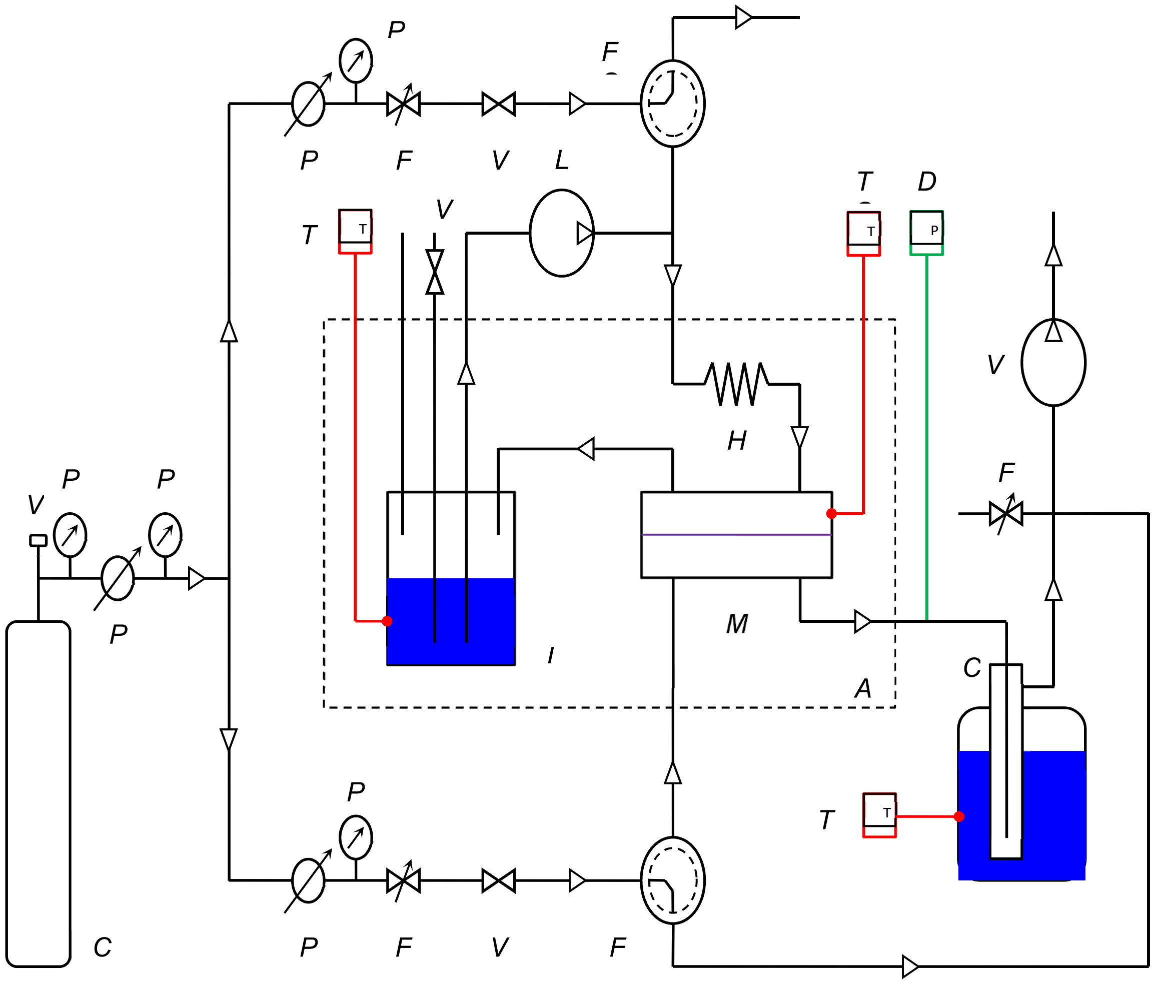

Figure S1. Pervaporation laboratory setup: AT – air thermostat, C – condenser, CG – carrier gas, D – digital pressure gauge, FC – flow controller, FS – flow switch, HE – heat exchanger, LP – liquid pump, LV – liquid vessel, MC – membrane cell, PG – pressure gauge, PR – pressure regulator, TS – temperature sensor, V – valve, VP – vacuum pump.

An isothermal experimental study of pervaporation involving separation of the water/ethanol mixtures using Romakon-PM 102 membrane was carried out using a laboratory setup shown in Fig. 1. The initial ethanol concentration in the model aqueous solution was 8.7 wt%, and the initial amount of model solution was 60 g. effective membrane area in the cell was 25.5 cm^2, the absolute permeate pressure was measured to be 2±1 kPa, permeate vapours were condensed in a trap cooled down to 6 °C. Membrane cell with a liquid vessel, heat exchanger and pipelines were placed in an air thermostat to keep isothermal conditions in the system and prevent condensation of vapours, experiment was carried out at a temperature of 68.8 °C. The feed solution flow rate was 2.5 cm^3/s. The sampling of feed solution was carried out periodically using an additional tap from the liquid vessel. The composition of the initial solution and samples was analyzed using gas chromatograph Shimadzu GC-8A, the analysis parameters were as follows: injector temperature 150 °C, packed column (1 m x 3 mm) with Chromosorb 102 sorbent, column temperature 150 °C, TCD current 80 mA.
